# Effect of second iron injection on growth performance, hematological parameters, and fecal microbiome of piglets fed different dietary iron levels

**DOI:** 10.1093/jas/skae371

**Published:** 2024-12-07

**Authors:** Alaina J Johnson, Wenli Li, Barbara I Dittrich, Aleah C Cole, Marie K Prodell, J Wesley Lyons, Scott A Fritz, Priscila Fregulia, Chi Chen, Chan Ho Kwon, Young Dal Jang

**Affiliations:** Department of Animal and Food Science, University of Wisconsin-River Falls, River Falls, WI 54022, USA; United States Department of Agriculture – Agricultural Research Service, US Dairy Forage Research Center, Madison, WI, 53706, USA; Department of Animal and Food Science, University of Wisconsin-River Falls, River Falls, WI 54022, USA; Department of Animal and Food Science, University of Wisconsin-River Falls, River Falls, WI 54022, USA; Department of Animal and Food Science, University of Wisconsin-River Falls, River Falls, WI 54022, USA; Pharmacosmos Inc., Watchung, NJ 07069, USA; Kansas State Veterinary Diagnostic Laboratory, Kansas State University, Manhattan, KS 66506, USA; United States Department of Agriculture – Agricultural Research Service, US Dairy Forage Research Center, Madison, WI, 53706, USA; Oak Ridge Institute for Science and Education, Oak Ridge, TN 37830, USA; Department of Animal Science, University of Minnesota, St. Paul, MN 55108, USA; Department of Animal and Dairy Science, University of Georgia, Athens, GA 30602, USA; Department of Animal and Food Science, University of Wisconsin-River Falls, River Falls, WI 54022, USA; Department of Animal and Dairy Science, University of Georgia, Athens, GA 30602, USA

**Keywords:** dietary iron level, fecal microbiome, growth, hemoglobin, iron injection, piglets

## Abstract

This experiment was conducted to evaluate the effects of a second iron injection for suckling pigs fed diets with different dietary iron levels in the nursery period on growth performance, hematological parameters, serum and liver trace mineral content, fecal score, microbiome, and metabolites. A total of 70 newborn pigs from 7 litters were assigned to either 1 or 2 iron injections within the litter and received the first i.m. iron injection (200 mg) at 2 to 3 d of age. Pigs assigned to the second injection treatment received an additional iron injection 5 d after the first injection. At weaning (days 27 to 30 of age), pigs within iron injection treatments were divided into 2 nursery diet treatments for a 27-d growth period. Treatments were 1) no additional iron injection + nursery diets with 100 ppm iron (NC), 2) second i.m. iron injection (200 mg) + NC diets, 3) no additional iron injection + nursery diets with 200 ppm iron (PC), and 4) second i.m. iron injection (200 mg) + PC diets. The second iron injection increased liver iron content at weaning (*P* = 0.08, tendency), and serum iron, hemoglobin, and hematocrit levels until day 13 postweaning (*P* < 0.05). In the nursery period, pigs receiving the second iron injection had a greater final body weight (*P* = 0.08, tendency), overall growth rate (*P* = 0.08, tendency) and feed intake (*P* < 0.05), and lower fecal score (*P* < 0.05) indicating firmer feces compared to those receiving 1 iron injection. There was no major effect of dietary iron level or interaction with the iron injection treatment in any measurements except that the pigs fed the PC diets had greater hemoglobin and hematocrit levels (*P *< 0.05) at day 27 postweaning and a lower fecal score (*P* = 0.08, tendency) in the late nursery period than those fed the NC diets. The second iron injection reduced fecal bacterial alpha-diversity based on Faith’s phylogenetic diversity at weaning (*P* < 0.05), while the second iron injection and dietary iron levels resulted in dissimilarity in the fecal bacterial community based on Unweighted Unifrac analysis (*P* < 0.05; at weaning by iron injection and day 27 postweaning by dietary iron level). In conclusion, the second iron injection for suckling pigs improved postweaning growth performance and hemoglobin levels and affected the fecal microbiome, whereas an additional 100 ppm of dietary iron supplementation increased hemoglobin levels and altered the fecal microbiome in the late nursery period but did not affect postweaning growth.

## Teaser

This study evaluated the potential effects of a second iron injection given to suckling pigs on growth, blood, and fecal parameters of pigs fed nursery diets with different dietary iron levels. The results showed that the second iron injection given at 7 to 8 d of age could enhance hemoglobin status and postweaning growth performance of pigs and alter fecal bacterial community, while dietary iron levels had no effect on growth performance.

## Introduction

Iron injection at birth is a common practice to prevent piglets from developing iron deficiency anemia during the suckling period in pig production (Chevalier et al., 2021), because newborn piglets are susceptible to iron deficiency due to insufficient iron reserves at birth and a low level of iron in sow milk (Matte and Audet, 2020). However, with increasing weaning age up to 28 d and a fast growth rate of suckling pigs, a single iron injection may not be sufficient to meet the iron needs of pigs for the entire suckling period as hemoglobin and hematocrit levels could decline from the third week of the suckling period (Perri et al., 2015; Chevalier et al., 2021; Friendship et al., 2021). Therefore, an additional iron injection during the suckling period may be needed for pigs to meet their iron demands for hemoglobin synthesis and immune and physiological functions which can impact growth in the suckling and nursery periods.

Iron administration to piglets via iron injection and dietary supplementation after weaning may be closely related because body iron status affects iron absorption from the gut. Increased body iron levels induce hepcidin expression that reduces iron absorption (Lipiński et al., 2010; Chen et al., 2019). Although the NRC (2012) recommended dietary iron concentrations of 100 ppm for nursery pigs, over 100 ppm of iron have been additionally supplemented to most swine diets (Faccin et al., 2023). It has been assumed that additional iron supplementation may be needed to prevent pigs from experiencing an excessive decline in iron stores after weaning because the bioavailability of intact iron in the feed ingredients is low (Rincker et al., 2004). However, as common mineral sources used in swine diets like calcium phosphate and limestone have relatively higher iron content (NRC, 2012) compared with other feed ingredients, dietary iron requirements could be met when these typical feed ingredients are used (Rincker et al., 2004). In addition, depending on dietary iron level and body iron status, a large amount of dietary iron may remain in the intestine, thus facilitating the proliferation of pathogenic bacteria such as *E. coli* and *Salmonella* causing postweaning diarrhea as these bacteria require iron for their survival and growth (Guo et al., 2017; Domínguez-Acuña and García-del Portillo, 2022). Also, high free iron content in the gut could increase oxidative stress as iron itself acts as a prooxidant (Li et al., 2016). Therefore, improving body iron status before weaning with an additional iron injection could reduce dietary iron requirements, which may result in an improved gut environment and reduced oxidative stress in pigs. There is limited information about the effectiveness of the second iron injection given during the suckling period in combination with dietary iron levels for nursery pigs. Investigating the potential to reduce iron levels in nursery diets via the second iron injection during the suckling period, while improving hematological status and postweaning growth represents an important data gap. It is also important to demonstrate how this iron injection strategy influences the growth and health of nursery pigs as newly weaned pigs are susceptible to postweaning disease infection due to an underdeveloped digestive system.

The aim of this study was to concurrently evaluate the effects of second iron injection and dietary iron levels on growth performance, hematological parameters, serum and liver mineral content, fecal score, short-chain fatty acids, and the microbiome of pigs.

## Materials and Methods

### Animal care

All procedures used in this study were approved by the Institutional Animal Care and Use Committee of University of Wisconsin-River Falls (Protocol # 20-21-56350). The experiment was conducted in the nursery facility at Mann Valley Farm of University of Wisconsin-River Falls (WI, USA).

### Animals, housing, and treatments

A total of 70 newborn piglets [initial body weight (**BW**): 1.83 ± 0.31 kg; Yorkshire × Yorkshire, Yorkshire × Duroc, Yorkshire × Duroc × Duroc; 40 males and 30 females] from 7 lactating sows with 8 to 12 piglets per litter were allotted into 4 treatments within the litter in a 2 × 2 factorial design (main factors were the number of iron injection and dietary iron level in nursery diets) based on BW and sex and housed in the farrowing crate along with the sow, with a heat lamp and mat provided. At days 2 to 3 of age (day 0 of the experiment), all piglets received the first intramuscular (i.m.) injection of 200 mg iron and were processed based on the standard protocol of UWRF Mann Valley Farm in which male piglets were castrated on day 10 of age. Piglet sets were created by having 4 littermates within the same sex that had similar BW. Within each set, 2 piglets were assigned to the 1 iron injection treatment and the others to the second iron injection treatment. Treatments were: 1) Negative control (**NC**): No additional iron injection + NC diets (100 mg/kg iron) in nursery period, 2) IRON + NC: Second i.m. iron injection (200 mg) at 5 d after the first injection + NC diets in nursery period, 3) Positive control (**PC**): No additional iron injection + PC diets (200 mg/kg iron) in nursery period, and 4) IRON + PC: Second i.m. iron injection (200 mg) at 5 d after the first injection + PC diets in nursery period. A common iron-dextran product (UNIFERON 200, Pharmacosmos, Inc., Watchung, NJ) was used for both injections. The piglets in the second iron injection treatments (IRON + NC and IRON + PC) received an additional 200 mg of i.m. iron injection at 5 d after the first injection (day 5 of the experiment) and all piglets were weaned at day 25 of the experiment (days 27 to 30 of age). There was no cross-fostering among litters and no creep feed was provided to the piglets during the entire suckling period. At weaning, all remaining piglets after tissue collection were moved to the nursery facility, mixed, and sorted by BW and sex to avoid sow effects. Then, the pigs within iron injection treatments were divided into nursery diet treatments in 3 replicate pens per treatment balanced by BW and sex with 5 or 6 pigs per pen for a 27-d postweaning growth period. The piglets were housed in raised-deck nursery pens (1.32 m × 1.63 m) with plastic or woven-wire flooring in an environmentally controlled nursery facility and had free access to water and feed. After weaning, piglets tested positive for hemolytic *E. coli* strains and *Rotaviral enteritis*. This was confirmed as fresh feces were collected at days 6 and 13 postweaning and analyzed at the Iowa State University Veterinary Diagnostic Laboratory (ISU-VDL, Ames, IA).

### Experimental diets

Corn–soybean meal-based basal diets with monocalcium phosphate and limestone were formulated to meet or exceed the NRC (2012) nutrient requirement estimates for nursery pigs weighing 7 to 25 kg except for iron for a 27-d feeding trial in the nursery period with 2 diet phases (Phase I and II: days 0 to 13 and 14 to 27 postweaning, respectively; Table 1). All major ingredients (corn, soybean meal, fish meal, whey, monocalcium phosphate, and limestone) were analyzed for iron content at the University of Missouri Agricultural Experiment Station Chemical Laboratories (Colombia, MO) before diet formulation, and iron values in each ingredient were applied to the diet formulation. For mixing experimental diets, a single batch of the basal diet was mixed without iron to prevent differences in the non-treatment components of the diets, and the basal diet contained approximately 85 ppm of iron (84.94 and 84.18 mg/kg for phases 1 and 2, respectively). Then, the basal diet was divided into 2 fractions. One fraction was mixed with an additional 15 ppm of iron for NC diets, resulting in 100 mg/kg of iron that meets the iron requirement (100 mg/kg) for nursery pigs weighing 7 to 25 kg based on NRC (2012) nutrient requirement estimates. An additional 115 mg/kg of iron was added to the other fraction for PC diets, resulting in 200 mg/kg of iron, which contains an additional 100 mg/kg of iron over the iron requirement in NRC (2012). Ferrous sulfate monohydrate was used in all diets for dietary iron supplementation. Feed samples were collected from diet mixing and analyzed for proximate composition and iron content in duplicate at the University of Missouri Agricultural Experiment Station Chemical Laboratories (Colombia, MO; Table 2).

**Table 1.** Diet formulation and calculated chemical composition

Ingredients, %	Phase 1 (days 0 to 13 postweaning)	Phase 2 (days 13 to 27 postweaning)
Corn	46.62	55.15
Soybean meal (48% CP)	30.20	30.00
Fish meal	2.50	0.00
Whey, dried	15.00	10.00
Soybean oil	2.80	1.80
l-Lysine·HCl	0.32	0.33
dl-MHA^1^	0.21	0.18
l-Threonine	0.110	0.10
Limestone	0.94	1.08
Monocalcium phosphate	0.70	0.76
Salt	0.50	0.50
Vitamin premix^2^	0.05	0.05
Trace mineral premix^3^	0.05	0.05
Calculated chemical composition		
Metabolizable energy (kcal/kg)	3,442	3,379
Crude protein (%)	22.14	20.55
SID^4^ lysine (%)	1.35	1.23
SID methionine + cysteine (%)	0.77	0.70
SID threonine	0.82	0.75
Total Ca (%)	0.80	0.71
STTD^4^ P (%)	0.43	0.36
Fe content (mg/kg)^5^	84.94	84.18

^1^ dl-Methionine hydroxy analogue with 84% methionine and 12% calcium.

^2^The vitamin premix supplied the following per kilogram of diet: 11,011 IU of vitamin A, 1,652 IU of vitamin D_3_, 55.1 IU of vitamin E, 4.4 mg of vitamin K, 0.044 mg of vitamin B_12_, 9.9 mg of riboflavin, 60.6 mg of pantothenic acid, 55.1 mg of niacin, 1.1 mg of folic acid, 3.3 mg of vitamin B_6_, 3.3 mg of thiamin, and 0.2 mg of biotin.

^3^The trace minerals supplied the following per kilogram of diet without iron: 40 mg of manganese as manganese sulfate, 100 mg of zinc as zinc sulfate, 15 mg of copper as copper sulfate, 0.3 mg of iodine as calcium iodate, and 0.3 mg of selenium as sodium selenite.

^4^SID = standardized ileal digestible, STTD = standardized total tract digestible.

^5^Iron content of ingredients were analyzed before diet formulation and iron content in the basal diet with no iron source was calculated. Additional iron as ferrous sulfate monohydrate was added to the basal diets by replacing corn to obtain 100 mg iron/kg of NC diets (100.3 and 100.7 mg/kg for phases 1 and 2, respectively) and to obtain 200 mg iron/kg of PC diets (200.5 and 201.2 mg/kg iron in phases 1 and 2, respectively).

**Table 2.** Analyzed chemical composition of experimental diets^1^

	Phase 1		Phase 2	
Item	NC^2^	PC^3^	NC^2^	PC^3^
Dry matter, %	89.01	88.97	87.85	87.93
Crude protein, %	21.92	21.14	18.31	18.60
Ether extract, %	3.90	4.00	2.89	2.90
Crude fiber, %	1.97	1.99	2.09	1.95
Ash, %	6.67	6.24	5.49	5.92
Iron, mg/kg	135	195	140	227

^1^Diets were analyzed in duplicate for iron content.

^2^Negative control with 100 mg/kg iron in nursery diets.

^3^Positive control with 200 mg/kg iron in nursery diets.

### Data and sample collection

In the suckling period, the BW of each pig was recorded at initial (day 0 of the experiment), days 5, 11, 18, and 25 (weaning) of the experiment. Blood samples from all pigs were collected from the jugular vein into K_3_ EDTA tubes each day when BW was recorded for hematocrit analysis. A drop of blood from the ear veins of the pigs was loaded into disposable microcuvettes via capillary action and analyzed for hemoglobin levels.

At weaning, 3 gilts per iron injection treatment that had average BW within the iron injection treatment were selected and euthanized by penetrating captive bolt by trained personnel, followed by exsanguination for liver sample collection. Collected liver samples were flash-frozen in liquid nitrogen and stored at −80 °C until the trace mineral analysis.

In the nursery period, BW of each piglet and pen feed consumption were recorded at days 0, 6, 13, 20, and 27 postweaning for calculation of average daily gain (**ADG**), average daily feed intake (**ADFI**), and gain-to-feed ratio (G:F). The fecal score was recorded every day for the entire experimental period using a 4-scale fecal score system (1 = normal, 2 = soft, looser than normal feces, slight diarrhea, 3 = moderate diarrheic feces, and 4 = liquid, severe diarrhea) by observing individual piglets in each pen and assessing signs of stool consistency in the pen. Blood samples from 6 representative piglets (1 barrow and 1 gilt per pen having average BW within each pen) were collected from jugular vein into K_3_ EDTA tubes at weaning, days 6, 13, and 27 postweaning, and analyzed for complete blood count. Blood samples were also collected into vacutainer trace element blood collection tubes with serum clot activator, and serum samples were separated by centrifugation at 2,500 × *g* for 25 min at 4 °C and stored at −80 °C until the analysis. Six serum samples from each treatment were analyzed for trace mineral content.

Fecal samples were collected by rectal palpation from 6 pigs (3 barrows and 3 gilts) per iron injection treatment at weaning, and from 2 representative pigs (average BW used for blood sampling) in each pen at days 6 and 27 postweaning, flash-frozen in liquid nitrogen, and stored at −80 °C until fecal microbiome and short-chain fatty acid analysis (**SCFA**).

### Sample analysis

Blood was analyzed for hemoglobin and hematocrit levels using the HemoCue Hb 201 + analyzer (HemoCue America, Brea, CA) and hematocrit microcentrifuge, respectively, at initial (day 0 of the experiment), days 5, 11, 18, and 25 of the experiment. The microcuvette with loaded blood was placed into the HemoCue Hb 201+, and the hemoglobin level was recorded. For hematocrit level determination, two 75-mm sodium-heparinized capillary tubes (Jorgensen Laboratories Inc., Loveland, CO) were filled with blood from each blood sample and then packed with clay prior to being put in the microhematocrit centrifuge (UNICO, Dayton, NJ). After the samples were centrifuged at 14,837 × *g* for 6 min and the plasma and red blood cells were separated, the capillary tubes were placed on a microhematocrit reader (Jorgensen Laboratories Inc., Loveland, CO), and the hematocrit level was determined in duplicate by 2 trained observers.

At weaning, days 6, 13, and 27 postweaning, complete blood count analysis including hemoglobin, hematocrit, red blood cell, white blood cell, mean corpuscular volume (**MCV**), mean corpuscular hemoglobin (**MCH**), mean corpuscular hemoglobin concentration (**MCHC**), platelet count, segmented neutrophils, lymphocytes, monocytes, eosinophils, bands (immature neutrophils), plasma protein, and fibrinogen was performed by the ADVIA 2120i Hematology System (Siemens Healthcare Diagnostics, Tarrytown, NY) at the South Dakota State University Animal Disease Research & Diagnostic Laboratory (Brookings, SD).

Collected serum and liver samples were analyzed for trace mineral content at the Kansas State Veterinary Diagnostic Laboratory (Manhattan, KS). Briefly, 1 g of the sample was digested in concentrated nitric acid (1.5 mL) and 30% hydrogen peroxide (0.5 mL—liver only) or concentrated nitric acid (1.5 mL) and 18Ω water (1.5 mL—serum only) via microwave digestion (Multiwave Pro, Anton Paar, Ashland, VA, USA). The microwave power was 1,500 W, and the following temperature program was used for digestion: from 0 to 15 min, the temperature was ramped to 165 °C then held at 165 °C for 15 min before cooling down to 70 °C in 16 min. Following digestion, the volume of the digested sample was adjusted to 10 mL with 18Ω water. Additionally, a 1:1 dilution was prepared with 18Ω water before analysis. A method blank containing all reagents, and no biological material was included in the run. Samples were diluted after digestion with 18Ω water prior to analysis via inductively coupled plasma mass spectrometry (ICP-MS; NexIon 350, Perkin Elmer, Shelton, CT). An 8-point calibration curve was built using standards in 2% aqueous nitric acid. The concentrations for the elements Cd, Co, Cr, Mn, Mo, Ni, Se, and zinc ranged from 0.00064 ppm to 1 ppm. The concentrations for the elements Ca, Cu, Fe, K, Mg, Na, and P ranged from 0.0064 ppm to 10 ppm. An internal standard solution containing Yttrium and Germanium was infused into each run (standards, samples, blanks).

Fecal samples were prepared by mixing with 50% aqueous acetonitrile containing deuterated acetate (d4-acetate) as internal standard in a 1:10 (w/v) ratio and then centrifuging at 18,000 × *g* for 10 min to obtain fecal extract supernatants. To detect SCFA, fecal extracts were first derivatized using 2-hydrazinoquinoline (Lu et al., 2013). The derivatized fecal extracts were separated using a BEH C18 column (Waters, Milford, CT) in an Acquity ultraperformance liquid chromatography system (Waters) and then detected in a Xevo-G2-S quadrupole time of flight mass spectrometer (**QTOFMS**) system (Waters). The conditions of LC-MS analysis, including mobile phase, and the parameters of MS detection have been described previously (Hung et al., 2022). Mass chromatograms and mass spectral data were acquired and processed using the MassLynx software V4.2 (Waters) in a centroided format. The concentrations of individual SCFA were determined by calculating the ratio between the peak area of the compound and the peak area of the internal standard and fitting with a standard curve using the Quanlynx software V4.2 (Waters).

### DNA extraction, library preparation, and sequencing

Total DNA was extracted from 250 mg of swine feces using the Qiagen RNeasy Power Microbiome kit according to the manufacturer’s instructions (Qiagen, Germany). DNA quantity was checked using the Qubit dsDNA assay (Q32851, Thermo Fisher, Waltham, MA). A xGen Amplicon 16S panel library kit (IDT, USA) was used to construct amplicon sequencing library preparation. This kit targets the V1–V9 region of the 16S rRNA molecular marker. For each sample, 35 ng of total DNA were used as the input. All libraries were prepared according to the manufacturer’s instructions, with modifications to the multiplex PCR thermal cycler program as follows: 98 °C for 30 s; 10 cycles of 98 °C for 10 s; 63 °C for 5 min; 65 °C for 1 min; 24 cycles of 98 °C for 10 s; 64 °C for 1 min; 65 °C for 1 min; 4 °C hold. The concentration of prepared libraries was calculated using a Kapa Kit (KK4873, Kapa Systems, Roche, Switzerland) with the RT-qPCR cycles run on a QuantStudio 5 instrument (Thermo Fisher, Waltham, MA) following the manufacturer’s instructions. An expected fragment size of 475 bp was included in the calculation. Library pooling and normalization were done in 2 steps: first, the initial library pooling was prepared using the pooling calculator by Illumina (https://support.illumina.com/help/pooling-calculator/pooling-calculator.htm, accessed on July 20, 2023) using the library concentration calculated using the Kapa Kit; second, pooled libraries were sequenced on a MiSeq using a Nano 300 cycle kit (Illumina Inc., San Diego, CA) to generate the individual index ratio for each sample in the pool. According to the index ratios obtained, the pooling volume of each sample was further normalized to ensure equal depth of sequencing. Finally, pooled libraries were sequenced on a MiSeq platform to obtain 2 × 150 bp paired-end reads for each sample (Illumina Inc., San Diego, CA).

### Statistical analysis and amplicon sequencing data analysis

All data were analyzed with ANOVA for a randomized complete block design using PROC MIXED of SAS (version 9.2; SAS Inst. Inc., Cary, NC). The experimental unit was an individual pig for growth performance in the suckling period, blood and fecal data, and a pen for growth performance and fecal score data in the nursery period. For the data from the suckling period, models included the treatment as a fixed effect and the sex within the litter, litter, and litter × treatment as random effects. For the data from the nursery period, models included the iron injection and dietary iron treatments and their interaction as fixed effects and the replication as a random effect. Initial BW was used as a covariate for growth performance due to the low *P*-value (*P* = 0.15) observed after allotting weanling pigs to nursery pens because the pigs were blocked by BW. Outlier analysis within each treatment and day was performed using the Grubb’s test outlier calculator (GraphPad Software, San Diego, CA), and no outlier was identified. Least squares means were separated using the PDIFF option of SAS. Multiple comparisons were adjusted using Tukey’s method. Statistical differences were considered significant at *P* < 0.05 and tendency at *P* < 0.10.

Microbial sequence data were analyzed using Quantitative Insights Into Microbial Ecology 2 (**QIIME2**) version 2021.11 (Bolyen et al., 2019), following the default method on the QIIME2 website. In brief, the data were demultiplexed and the reads were quality-filtered using the Divisive Amplicon Denoising Algorithm (DADA2) plugin implemented in QIIME2. The sequences were merged, and chimeric sequences were removed before the generation of a table of amplicon sequencing variants (**ASV**; Callahan et al., 2016). Representative sequences were aligned to the SILVA (Silva 138, 99% OTU full-length sequences). To analyze microbial diversity, sequence counts were standardized by rarefying them to the same number of sequences (the smallest sampling size). To investigate the alpha-diversity metrics, Pielou’s evenness, Shannon’s entropy, and Faith’s phylogenetic diversity were calculated. To investigate the beta-diversity metrics, the Bray–Curtis dissimilarity index, Jaccard index, and weighted UniFrac distance were calculated. Dissimilarity and distance between the fecal microbiota and the variables of the study were tested using Unweighted UniFrac distance matrices. One fecal sample per iron injection treatment at weaning was removed from the data analysis due to low DNA concentrations.

## Results

There were no differences in preweaning growth performance between 1- and 2-iron injection treatments in the suckling period (*P* > 0.10; Table 3). The second iron injection significantly increased hemoglobin (*P* < 0.05) and hematocrit (*P* < 0.05) levels in the suckling period from days 11 to 25 of the experiment. In complete blood count at weaning (Table 4), the second iron injection increased red blood cell count (*P* < 0.10, tendency), MCV (*P* < 0.05), and MCH (*P* < 0.05), but decreased platelet count (*P* < 0.10, tendency) and plasma protein level (*P* < 0.05). There was no difference in serum trace mineral level at weaning (Table 5), while the second iron injection increased calcium (*P* < 0.10, tendency) and iron (*P* < 0.10, tendency) content in the liver at weaning.

**Table 3.** Preweaning growth performance, hemoglobin and hematocrit levels of pigs receiving 1 or 2 iron injections in the suckling period^1^

	Treatment^2^			
Item	1 injection	2 injections	SEM	*P*-value
Body weight, kg				
Day 0	1.84	1.83	0.07	0.79
Day 5	3.19	3.18	0.12	0.96
Day 11	5.04	5.13	0.17	0.60
Day 18	7.21	7.33	0.20	0.61
Day 25 (weaning)	9.61	9.81	0.25	0.52
Hemoglobin, g/dL				
Day 5	8.8	8.6	0.40	0.54
Day 11	11.1	11.7	0.53	0.05
Day 18	10.3	12.2	0.34	<0.0001
Day 25 (weaning)	10.1	12.4	0.47	<0.01
Hematocrit, %				
Day 5	31.4	31.1	1.03	0.68
Day 11	35.3	37.6	1.28	<0.01
Day 18	32.8	38.2	0.96	<0.01
Day 25 (weaning)	31.7	37.9	1.06	<0.0001

^1^ *n* = 35 per treatment from all pigs used in the experiment.

^2^One-iron injection: 200 mg iron injection at 2 to 3 d of age (day 0 of the experiment) and no additional iron injection in the suckling period. Two-iron injection: 200 mg iron injections at 2 to 3 d of age and 5 d after the first injection (day 5 of the experiment).

**Table 4.** Complete blood count analysis for pigs receiving 1 or 2 iron injections in the suckling period at day 25 of the experiment (weaning)^1^

Item^3^	Reference range^4^	Treatment^2^			
		1 injection	2 injections	SEM	*P*-value
WBC, 10^3^/µL	11 to 22	9.14	8.44	1.15	0.65
RBC, 10^6^/µL	5 to 8	5.72	6.18	0.18	0.08
Hemoglobin, g/dL	10 to 16	9.72	11.74	0.42	0.01
Hematocrit, %	32 to 50	34.13	40.21	1.28	0.01
MCV, fl	50 to 68	59.64	66.40	1.76	0.03
MCH, pg	17 to 21	17.05	19.42	0.74	0.04
MCHC, g/dL	30 to 34	28.53	29.19	0.48	0.38
Platelet, 10^3^/µL	200 to 500	479.1	303.5	56.7	0.06
Segmented neutrophils, %	28 to 47	44.41	40.87	2.66	0.35
Lymphocytes, %	39 to 62	52.56	55.34	2.83	0.44
Monocytes, %	2 to 10	1.37	1.81	0.48	0.36
Eosinophils, %	0.5 to 11	1.33	0.91	0.34	0.42
Bands, %	0 to 4	0.48	0.97	0.29	0.24
Segmented neutrophils, /µL	2,000 to 15,000	4,311	3,467	723	0.45
Lymphocytes, /µL	3,800 to 16,500	4,578	4,615	481	0.94
Monocytes, /µL	0 to 1,000	121.9	164.1	44.4	0.31
Eosinophils, /µL	0 to 1,500	83.97	71.30	21.94	0.70
Bands, /µL	0 to 800	39.89	94.07	29.57	0.21
Plasma protein, g/dL	6 to 8	6.61	6.24	0.18	0.04
Fibrinogen, mg/dL	200 to 400	255.6	154.6	45.5	0.18

^1^ *n* = 12 per treatment from 2 pigs per pen at weaning.

^2^One-iron injection: 200 mg iron injection at 2 to 3 d of age and no additional iron injection in the suckling period. Two-iron injection: 200 mg iron injections at 2 to 3 d of age and 5 d after the first injection.

^3^WBC, white blood cell; RBC, red blood cell; MCV, mean corpuscular volume; MCH, mean corpuscular hemoglobin; MCHC, mean corpuscular hemoglobin concentration.

^4^From the Merck Veterinary Manual (2016).

**Table 5.** Serum and liver trace mineral content of pigs receiving 1 or 2 iron injections in the suckling period at day 25 of the experiment (weaning)^1^

	Treatment^2^			
Item	1 injection	2 injections	SEM	*P*-value
Serum, ppm				
Ca	60.91	63.30	2.14	0.36
Co, ppb^3^	3.30	1.59	0.90	0.16
Cr, ppb	3.96	2.63	0.95	0.23
Cu	1.45	1.41	0.09	0.47
Fe	1.65	2.27	0.30	0.16
K	163.0	167.0	7.2	0.70
Mg	21.18	21.67	0.70	0.63
Mn, ppb	7.75	6.18	0.92	0.20
Mo, ppb	18.44	15.87	5.69	0.62
Na	2,933	3,052	88	0.27
P	164.3	165.7	5.4	0.84
Se	0.12	0.12	0.01	0.85
Zn	0.76	0.77	0.05	0.70
Liver, mg/kg DM basis				
Ca	175.8	248.3	48.0	0.06
Co	0.06	0.07	0.01	0.23
Cr	0.18	0.26	0.05	0.44
Cu	202.8	191.9	61.7	0.82
Fe	373	1,424	250	0.08
K	10,826	11,993	730	0.35
Mg	686.6	798.6	45.7	0.15
Mn	10.88	11.01	1.81	0.95
Mo	1.55	1.82	0.18	0.24
Na	5,261	5,413	917	0.92
P	11,839	13,338	715	0.12
Se	2.77	2.45	0.22	0.35
Zn	208.0	259.2	63.9	0.43

^1^ *n* = 12 per treatment for serum; *n* = 3 per treatment for liver.

^2^One-iron injection: 200 mg iron injection at 2 to 3 d of age and no additional iron injection in the suckling period. Two-iron injection: 200 mg iron injections at 2 to 3 d of age and 5 d after the first injection (day 5 of the experiment).

^3^ppb unit is used due to low concentrations detected.

In the postweaning period (Table 6), the pigs in the 2-iron injection treatment tended to have greater final BW (*P* < 0.10, tendency) at days 27 postweaning and ADG in days 13 to 27 (*P* < 0.10 tendency) and 0 to 27 (*P* < 0.10, tendency) postweaning and had significantly greater ADFI in days 0 to 13 (*P* < 0.05), 13 to 27 (*P* < 0.05), and 0 to 27 (*P* < 0.05) postweaning than those in the 1-iron injection treatment, while the pigs in the 2-iron injection treatment had lower fecal score in days 13 to 27 (*P* < 0.05) and 0 to 27 (*P* < 0.05) postweaning than those in the 1-iron injection treatment. The dietary iron levels had no significant effect on growth performance (Table 6) except for the pigs fed the PC diets showing lower fecal score compared with those fed the NC diets in days 13 to 27 postweaning (*P* < 0.10, tendency).

**Table 6.** Postweaning growth performance and fecal score of pigs receiving 1 or 2 iron injections in the suckling period and different dietary iron levels in nursery period^1^

	Injection^2^		Dietary Fe^3^			*P*-value		
Item	1 injection	2 injections	100 ppm	200 ppm	SEM	Injection (I)	Dietary Fe (D)	I × D
Body weight, kg								
Day 0	9.71	9.91	9.77	9.84	0.67	0.15	0.57	0.97
Day 13	11.09	11.76	11.54	11.31	0.29	0.16	0.60	0.66
Day 27	18.84	20.75	20.02	19.58	0.60	0.08	0.62	0.35
ADG, kg/d								
Days 0 to 13 (phase I)	0.099	0.150	0.133	0.116	0.022	0.16	0.61	0.66
Days 13 to 27 (phase II)	0.554	0.642	0.606	0.590	0.028	0.08	0.71	0.28
Days 0 to 27 (overall)	0.335	0.405	0.378	0.362	0.022	0.08	0.62	0.35
ADFI, kg/d								
Days 0 to 13 (phase I)	0.287	0.348	0.329	0.307	0.013	0.02	0.29	0.84
Days 13 to 27 (phase II)	0.923	1.033	0.999	0.957	0.028	0.04	0.35	0.18
Days 0 to 27 (overall)	0.617	0.703	0.676	0.644	0.019	0.03	0.29	0.27
G:F								
Days 0 to 13 (phase I)	0.334	0.426	0.394	0.365	0.066	0.37	0.77	0.88
Days 13 to 27 (phase II)	0.601	0.623	0.609	0.615	0.024	0.56	0.86	0.88
Days 0 to 27 (overall)	0.542	0.576	0.559	0.559	0.028	0.43	0.99	0.75
Fecal score								
Days 0 to 13 (phase I)	2.40	2.23	2.31	2.33	0.08	0.19	0.88	0.55
Days 13 to 27 (phase II)	2.13	1.69	2.04	1.78	0.08	0.01	0.08	0.94
Days 0 to 27 (overall)	2.26	1.95	2.17	2.04	0.07	0.03	0.28	0.78

^1^ *n* = 3 replicate pens per treatment. Initial BW was used as a covariate in statistical analysis for growth performance.

^2^One-iron injection: 200 mg iron injection at 2 to 3 d of age and no additional iron injection in the suckling period. Two-iron injection: 200 mg iron injections at 2 to 3 d of age and 5 d after the first injection.

^3^NC: negative control with 100 mg/kg iron in nursery diets; PC: positive control with 200 mg/kg iron in nursery diets.

The second iron injection during the suckling period increased serum iron levels at days 6 and 13 postweaning (*P* < 0.05; Table 7), molybdenum and sodium levels at day 13 postweaning (*P* < 0.05), while decreasing copper and selenium levels at day 13 postweaning (*P* < 0.05) and cobalt at day 27 postweaning (*P* < 0.05). The PC diets increased serum potassium levels at day 6 postweaning (*P* < 0.10, tendency; Table 7), but decreased cobalt (*P* < 0.05) molybdenum (*P* < 0.10, tendency) levels at day 13 postweaning and cobalt, molybdenum, and phosphorus levels at day 27 postweaning (*P* < 0.05) compared with the NC diets.

**Table 7.** Serum trace mineral content (ppm) of pigs receiving 1 or 2 iron injections in the suckling period and different dietary iron levels in nursery period^1^

	Injection^2^		Dietary Fe^3^			*P*-value		
Item	1 injection	2 injections	100 ppm	200 ppm	SEM	Injection (I)	Dietary Fe (D)	I × D
Day 6 postweaning								
Ca	60.22	60.54	59.56	61.20	1.66	0.89	0.49	0.55
Co, ppb^4^	0.30	0.24	0.34	0.20	0.08	0.48	0.10	0.37
Cr, ppb	3.60	3.31	3.51	3.40	0.23	0.33	0.71	0.89
Cu	1.46	1.38	1.40	1.44	0.05	0.29	0.64	0.57
Fe	1.54	2.14	1.71	1.97	0.19	0.01	0.22	0.84
K	193.3	191.4	185.0	199.7	5.4	0.80	0.08	0.57
Mg	20.43	20.10	19.99	20.54	0.59	0.70	0.52	0.50
Mn, ppb	4.46	6.15	6.34	4.27	1.59	0.39	0.29	0.59
Mo, ppb	6.35	7.25	6.41	7.19	1.44	0.50	0.56	0.08
Na	2,900	3,059	2,946	3,013	68	0.12	0.49	0.68
P	99.6	105.0	101.1	103.6	2.8	0.18	0.53	0.20
Se	0.15	0.14	0.15	0.15	0.01	0.90	0.99	0.27
Zn	0.50	0.51	0.49	0.52	0.03	0.66	0.30	0.21
Day 13 postweaning								
Ca	62.63	65.41	64.16	63.88	1.32	0.13	0.87	0.66
Co, ppb	0.13	0.14	0.18	0.09	0.03	0.81	0.02	0.81
Cr, ppb	4.05	3.73	3.92	3.86	0.17	0.12	0.77	0.09
Cu	1.30	1.13	1.15	1.27	0.05	0.05	0.15	0.44
Fe	1.48	2.57	2.00	2.05	0.27	0.01	0.87	0.58
K	182.9	191.1	185.9	188.1	6.7	0.39	0.81	0.29
Mg	18.55	17.81	17.93	18.43	0.47	0.29	0.47	0.43
Mn, ppb	3.71	5.69	5.43	3.98	0.92	0.15	0.28	0.27
Mo, ppb	6.13	9.82	9.42	6.53	1.40	0.04	0.10	0.60
Na	3,057	3,199	3,124	3,132	48	0.05	0.91	0.23
P	108.2	102.3	105.7	104.7	3.3	0.23	0.83	0.91
Se	0.16	0.14	0.15	0.14	0.01	0.01	0.36	0.50
Zn	0.52	0.55	0.53	0.54	0.02	0.26	0.87	0.35
Day 27 postweaning								
Ca	67.25	67.01	68.72	65.54	2.12	0.92	0.21	0.82
Co, ppb	0.27	0.17	0.28	0.15	0.04	0.03	0.01	0.42
Cr, ppb	4.88	4.44	4.71	4.61	0.24	0.23	0.78	0.21
Cu	1.10	1.03	1.08	1.05	0.04	0.24	0.62	0.28
Fe	2.36	2.08	2.10	2.34	0.18	0.20	0.27	0.79
K	181.5	189.4	188.6	182.3	8.4	0.36	0.46	0.84
Mg	18.25	18.64	18.15	18.73	0.66	0.54	0.36	0.72
Mn, ppb	4.71	4.30	4.68	4.33	0.27	0.17	0.25	0.80
Mo, ppb	15.80	15.81	19.33	12.27	2.30	1.00	0.04	0.44
Na	3,044	3,088	3,052	3,079	75	0.63	0.77	0.81
P	104.3	106.8	110.5	100.7	3.0	0.56	0.04	0.09
Se	0.15	0.16	0.14	0.16	0.01	0.27	0.13	0.38
Zn	0.67	0.65	0.69	0.62	0.03	0.64	0.16	0.93

^1^ *n* = 6 replicate pens per treatment.

^2^One-iron injection: 200 mg iron injection at 2 to 3 d of age and no additional iron injection in the suckling period. Two-iron injection: 200 mg iron injections at 2 to 3 d of age and 5 d after the first injection.

^3^NC: negative control with 100 mg/kg iron in nursery diets; PC: positive control with 200 mg/kg iron in nursery diets.

^4^ppb unit is used due to low concentrations detected.

The second iron injection during the suckling period increased the levels of hemoglobin, hematocrit, MCV, MCH, MCHC, segmented neutrophil percentage (*P* < 0.05; Table 8) but decreased the platelet count and plasma protein level (*P* < 0.10, tendency), lymphocyte percentage, monocyte count and percentage (*P* < 0.05) in blood at day 6 postweaning. The PC diets increased segmented neutrophil percentage (*P* < 0.05) and tended to increase monocyte count and percentage and fibrinogen level (*P* < 0.10, tendency) but decreased the lymphocyte percentage (*P* < 0.05) in blood at day 6 postweaning compared with the NC diets. There was an interaction in fibrinogen level (*P* < 0.05) between iron injection and dietary iron level, where the PC diets increased fibrinogen level for the pigs in the 2-iron injection treatment while decreasing for those fed the NC diets.

**Table 8.** Complete blood count analysis of pigs receiving 1 or 2 iron injections in the suckling period and different dietary iron levels in nursery period (day 6 postweaning)^1^

	Injection^2^		Dietary Fe^3^			*P*-value		
Item^4^	1 injection	2 injections	100 ppm	200 ppm	SEM	Injection (I)	Dietary Fe (D)	I × D
WBC, 10^3^/µL	10.42	10.05	10.42	10.05	0.95	0.75	0.75	0.55
RBC, 10^6^/µL	6.42	6.79	6.58	6.64	0.17	0.15	0.81	0.33
Hemoglobin, g/dL	10.49	12.95	11.86	11.58	0.39	0.01	0.62	0.69
Hematocrit, %	37.71	44.05	41.18	40.58	1.19	0.01	0.73	0.60
MCV, fL	58.77	64.94	62.61	61.09	1.47	0.01	0.48	0.69
MCH, pg	16.34	19.12	18.04	17.42	0.53	0.01	0.43	0.63
MCHC, g/dL	27.77	29.46	28.75	28.48	0.31	0.01	0.55	0.84
Platelet, 10^3^/µL	529.0	323.9	404.6	448.3	68.5	0.06	0.66	0.34
Segmented neutrophils, %	40.83	48.89	42.40	47.33	1.83	0.01	0.05	0.11
Lymphocytes, %	55.86	49.62	55.68	49.80	1.94	0.02	0.03	0.16
Monocytes, %	1.76	0.46	0.69	1.53	0.32	0.01	0.08	0.50
Eosinophils, %	0.47	0.33	0.50	0.29	0.17	0.57	0.40	0.81
Bands, %	1.07	0.68	0.70	1.04	0.28	0.34	0.40	0.64
Segmented neutrophils, /µL	4,310	4,882	4,499	4,692	435	0.37	0.76	0.95
Lymphocytes, /µL	5,757	4,953	5,685	5,024	569	0.18	0.25	0.21
Monocytes, /µL	178.1	50.3	71.2	157.2	32.4	0.01	0.07	0.52
Eosinophils, /µL	53.83	37.32	59.73	31.42	19.36	0.56	0.32	0.71
Bands, /µL	113.9	78.2	74.8	117.3	34.8	0.48	0.41	0.62
Plasma protein, g/dL	6.69	6.26	6.54	6.41	0.17	0.08	0.57	0.81
Fibrinogen, mg/dL	395.9	396.4	347.4	444.8	46.0	0.99	0.10	0.01^5^

^1^ *n* = 6 per treatment.

^2^One-iron injection: 200 mg iron injection at 2 to 3 d of age and no additional iron injection in the suckling period. Two-iron injection: 200 mg iron injections at 2 to 3 d of age and 5 d after the first injection.

^3^NC: negative control with 100 mg/kg iron in nursery diets; PC: positive control with 200 mg/kg iron in nursery diets.

^4^WBC, white blood cell; RBC, red blood cell; MCV, mean corpuscular volume; MCH, mean corpuscular hemoglobin; MCHC, mean corpuscular hemoglobin concentration.

^5^Significant interaction between iron injection and dietary iron level, *P *< 0.05; means for the 1- and 2-iron injection treatments within NC and PC treatments were 441.75, 253.09, 350.00, and 539.65 mg/dL, respectively.

At day 13 postweaning, the second iron injection during the suckling period increased the levels of hemoglobin, hematocrit, MCV, MCH, and MCHC (*P* < 0.05; Table 9) but decreased platelet count (*P* < 0.05) and plasma protein level (*P* < 0.10, tendency) in the blood. The PC diets increased red blood cell, platelet count, plasma protein (*P* < 0.05), and monocyte percentage (*P* < 0.10, tendency) compared with the NC diets. There was an interaction in eosinophil count and percentage (*P* < 0.05) between iron injection and dietary iron level, where the PC diets increased eosinophil count and percentage for the pigs in the 2-iron injection treatment while decreasing for those fed the NC diets.

**Table 9.** Complete blood count analysis of pigs receiving 1 or 2 iron injections in the suckling period and different dietary iron levels in nursery period (day 13 postweaning)^1^

	Injection^2^		Dietary Fe^3^			*P*-value		
Item^4^	1 injection	2 injections	100 ppm	200 ppm	SEM	Injection (I)	Dietary Fe (D)	I × D
WBC, 10^3^/µL	16.44	15.96	16.78	15.62	0.80	0.68	0.32	0.58
RBC, 10^6^/µL	6.43	6.30	6.11	6.61	0.13	0.52	0.02	0.55
Hemoglobin, g/dL	9.92	11.45	10.51	10.86	0.29	0.01	0.41	0.78
Hematocrit, %	32.29	35.90	33.39	34.80	0.80	0.01	0.23	0.78
MCV, fL	50.54	57.05	54.91	52.68	1.33	0.01	0.24	0.73
MCH, pg	15.54	18.23	17.30	16.47	0.49	0.01	0.25	0.44
MCHC, g/dL	30.66	31.91	31.38	31.19	0.33	0.02	0.70	0.18
Platelet, 10^3^/µL	694.1	477.8	516.1	655.9	45.6	0.01	0.03	0.08
Segmented neutrophils, %	56.82	55.50	56.98	55.33	2.11	0.67	0.59	0.92
Lymphocytes, %	38.68	40.25	38.85	40.08	2.08	0.60	0.68	0.57
Monocytes, %	2.13	1.92	1.63	2.42	0.39	0.62	0.08	0.38
Eosinophils, %	1.42	1.42	1.58	1.25	0.28	1.00	0.35	0.01^5^
Bands, %	0.90	0.75	0.82	0.83	0.26	0.69	0.96	0.63
Segmented neutrophils, /µL	9,413	8,926	9,581	8,758	698	0.63	0.42	0.69
Lymphocytes, /µL	6,297	6,366	6,507	6,156	342	0.89	0.48	0.38
Monocytes, /µL	342.8	309.3	282.1	370	63.4	0.63	0.22	0.42
Eosinophils, /µL	234.0	224.6	263.4	195.2	47.7	0.88	0.29	0.01^5^
Bands, /µL	143.4	114.7	126	132.1	41.7	0.63	0.92	0.51
Plasma protein, g/dL	5.57	5.18	5.14	5.60	0.15	0.06	0.03	0.96
Fibrinogen, mg/dL	221.5	241.7	280.0	183.0	48.9	0.74	0.12	0.83

^1^ *n* = 6 per treatment.

^2^One-iron injection: 200 mg iron injection at 2 to 3 d of age and no additional iron injection in the suckling period. Two-iron injection: 200 mg iron injections at 2 to 3 d of age and 5 d after the first injection.

^3^NC: negative control with 100 mg/kg iron in nursery diets; PC: positive control with 200 mg/kg iron in nursery diets.

^4^WBC, white blood cell; RBC, red blood cell; MCV, mean corpuscular volume; MCH, mean corpuscular hemoglobin; MCHC, mean corpuscular hemoglobin concentration.

^5^Significant interactions between iron injection and dietary iron level, *P *< 0.05; means for 1- and 2-iron injection treatments within NC and PC treatments were 2.17%, 1.00%, 0.67%, and 1.83% and 354.80, 171.95, 113.20, and 277.28/µL, for eosinophil percentage and count, respectively.

At day 27 postweaning, the pigs in the 2-iron injection treatment had a greater level of MCV (*P* < 0.05; Table 10), while those tended to have a lower plasma protein level (*P* < 0.10, tendency) than the pigs in the 1-iron injection treatment. The high dietary iron level increased red blood cell count, hemoglobin, hematocrit levels, segmented neutrophil count and percentage (*P* < 0.05) but decreased lymphocyte (*P* < 0.05) and monocyte count and percentage (*P* < 0.10, tendency) in the blood. There were interactions between iron injection and dietary iron level in band count (*P* < 0.05) and eosinophil count and percentage (*P* < 0.05) in blood at day 27 postweaning, where the PC diets increased band count, eosinophil count, and percentage for the pigs in the 2-iron injection treatment while decreasing for those fed the NC diets. There were no differences in fecal short-chain fatty acid concentrations at day 27 postweaning (Table 11).

**Table 10.** Complete blood count analysis of pigs receiving 1 or 2 iron injections in the suckling period and different dietary iron levels in nursery period (day 27 postweaning)^1^

	Injection^2^		Dietary Fe^3^			*P*-value		
Item^4^	1 injection	2 injections	100 ppm	200 ppm	SEM	Injection (I)	Dietary Fe (D)	I × D
WBC, 10^3^/µL	16.89	16.39	16.34	16.94	1.12	0.76	0.71	0.30
RBC, 10^6^/µL	6.21	5.95	5.88	6.28	0.13	0.12	0.02	0.50
Hemoglobin, g/dL	10.61	10.85	10.40	11.06	0.16	0.31	0.01	0.17
Hematocrit, %	34.60	35.04	33.77	35.88	0.57	0.52	0.01	0.42
MCV, fL	55.93	59.04	57.71	57.26	1.05	0.05	0.77	0.21
MCH, pg	17.18	18.08	17.53	17.73	0.42	0.15	0.75	0.32
MCHC, g/dL	30.68	31.01	30.75	30.93	0.21	0.17	0.44	0.08
Platelet, 10^3^/µL	406.8	384.5	411.5	379.8	25.2	0.43	0.27	0.32
Segmented neutrophils, %	43.25	41.00	38.25	46.00	1.61	0.34	0.01	0.75
Lymphocytes, %	53.83	56.33	58.83	51.33	1.61	0.29	0.01	0.72
Monocytes, %	0.50	0.42	0.75	0.17	0.20	0.78	0.06	0.78
Eosinophils, %	1.08	1.33	1.08	1.33	0.34	0.61	0.61	0.04^5^
Bands, %	1.17	1.08	0.92	1.33	0.30	0.82	0.25	0.25
Segmented neutrophils, /µL	7,277	6,743	6,192	7,827	546.38	0.50	0.05	0.49
Lymphocytes, /µL	9,166	9,199	9,657	8,709	729.10	0.97	0.35	0.42
Monocytes, /µL	95.00	71.01	131.1	34.9	38.3	0.66	0.10	0.78
Eosinophils, /µL	158.3	289.8	180.5	267.6	57.5	0.12	0.29	0.01^5^
Bands, /µL	166.4	199.3	150.7	215.0	40.6	0.55	0.25	0.04^6^
Plasma protein, g/dL	5**.**99	5.66	5.77	5.88	0.15	0.09	0.54	0.60
Fibrinogen, mg/dL	275.0	233.3	216.7	291.7	77.6	0.62	0.38	0.49

^1^ *n* = 6 per treatment.

^2^One-iron injection: 200 mg iron injection at 2 to 3 d of age and no additional iron injection in the suckling period. Two-iron injection: 200 mg iron injections at 2 to 3 d of age and 5 d after the first injection.

^3^NC: negative control with 100 mg/kg iron in nursery diets; PC: positive control with 200 mg/kg iron in nursery diets.

^4^WBC, white blood cell; RBC, red blood cell; MCV, mean corpuscular volume; MCH, mean corpuscular hemoglobin; MCHC, mean corpuscular hemoglobin concentration.

^5^Significant interactions between iron injection and dietary iron level, *P *< 0.05; means for 1- and 2-iron injection treatments within NC and PC treatments were 1.50%, 0.67%, 0.67%, and 2.00% and 261.98, 99.08, 54.60, and 480.60/µL, for eosinophil percentage and count, respectively.

^6^Significant interaction between iron injection and dietary iron level, *P *< 0.05; means for 1- and 2-iron injection treatments within NC and PC treatments were 193.22, 108.15, 139.53, and 290.38/µL, respectively.

**Table 11.** Fecal SCFA of pigs receiving 1 or 2 iron injections in the suckling period and different dietary iron levels in nursery period (day 27 postweaning)^1^

	Injection^2^		Dietary Fe^3^			*P*-value		
Item^4^	1 injection	2 injections	100 ppm	200 ppm	SEM	Injection (I)	Dietary Fe (D)	I × D
Concentrations, µmol/g feces								
Acetic acid	238.00	229.40	244.02	223.38	18.33	0.72	0.39	0.72
Propionic acid	80.37	74.41	80.18	74.60	8.16	0.61	0.64	0.57
Butyric acid	52.75	45.51	52.12	46.15	5.77	0.38	0.47	0.87
Valeric acid	22.15	22.75	22.50	22.40	3.03	0.89	0.98	0.50
Isovaleric acid	5.86	4.49	4.67	5.68	1.05	0.32	0.45	0.28
Composition, % of total SCFA								
Acetic acid	60.55	61.62	61.51	60.66	1.44	0.61	0.68	0.25
Propionic acid	19.74	19.66	19.50	19.90	0.71	0.94	0.69	0.06
Butyric acid	12.92	11.81	12.52	12.22	0.66	0.25	0.75	0.80
Valeric acid	5.35	5.76	5.39	5.73	0.38	0.46	0.54	0.09
Isovaleric acid	1.43	1.15	1.08	1.50	0.18	0.28	0.13	0.21

^1^ *n* = 6 per treatment.

^2^One-iron injection: 200 mg iron injection at 2 to 3 d of age and no additional iron injection in the suckling period. Two-iron injection: 200 mg iron injections at 2 to 3 d of age and 5 d after the first injection.

^3^NC: negative control with 100 mg/kg iron in nursery diets; PC: positive control with 200 mg/kg iron in nursery diets.

**Figure 1.**
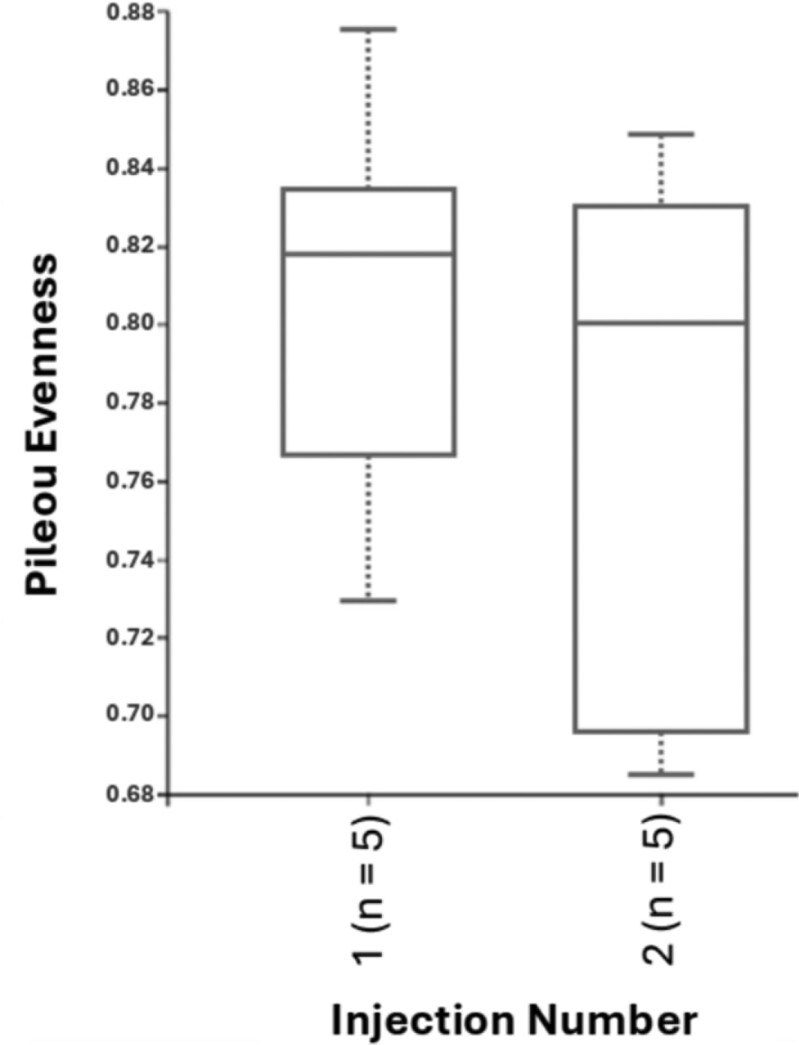
Alpha-diversity (evenness) index in feces of pigs at weaning (*P *> 0.10; *n* = 5 per injection treatment). One-iron injection (1): 200 mg iron injection at 2 to 3 d of age and no additional iron injection in the suckling period. Two-iron injection (2): 200 mg iron injections at 2 to 3 d of age and 5 d after the first injection. *n* in parenthesis of the figure represents the number of the samples.

**Figure 2.**
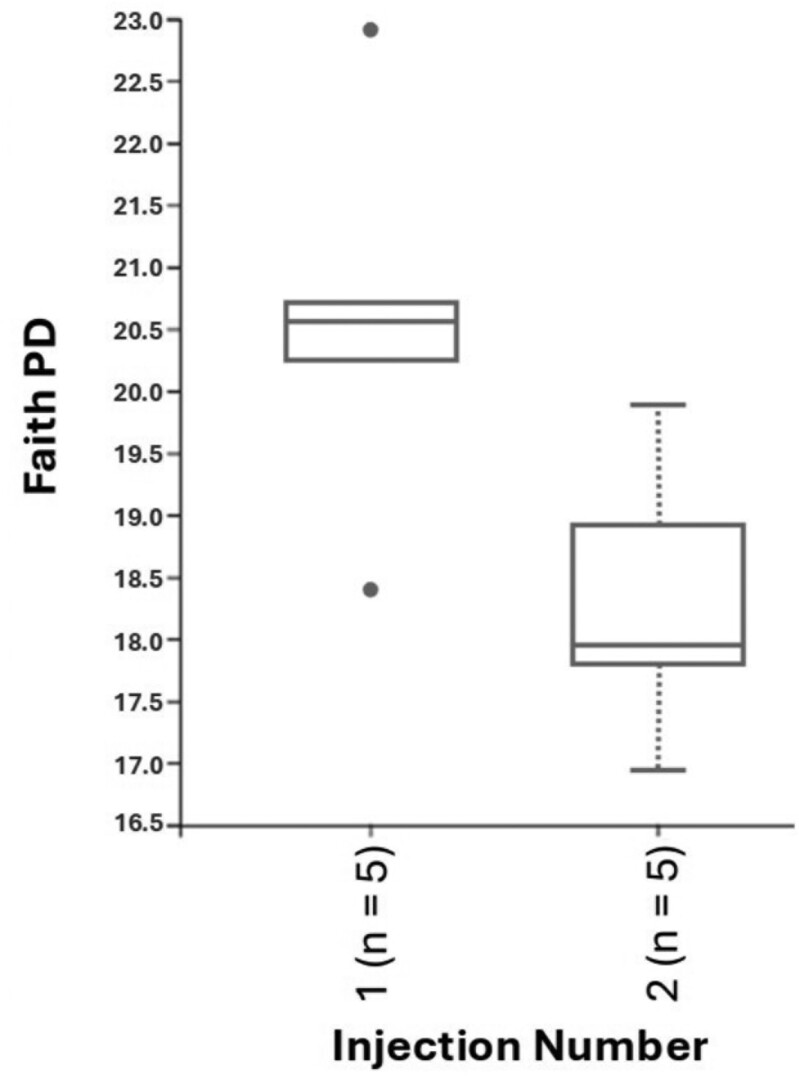
Alpha-diversity (Faith phylogenetic diversity index) in feces of pigs at weaning (*P *< 0.05; *n* = 5 per injection treatment). One-iron injection (1): 200 mg iron injection at 2 to 3 d of age and no additional iron injection in the suckling period. Two-iron injection (2): 200 mg iron injections at 2 to 3 d of age and 5 d after the first injection. *n* in parenthesis of the figure represents the number of the samples.

**Figure 3.**
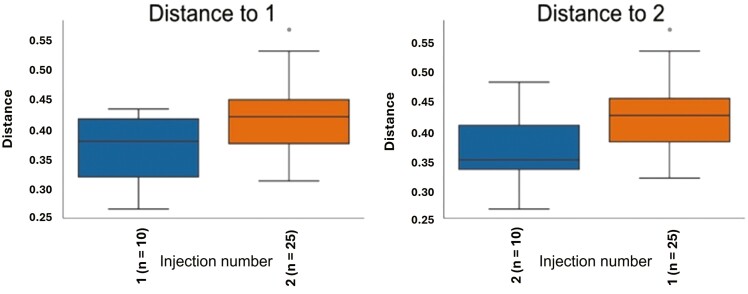
Beta-diversity (Unweighted Unifrac analysis) in feces of pigs at weaning (*P *< 0.05; *n* = 5 per injection treatment). One-iron injection (1): 200 mg iron injection at 2 to 3 d of age and no additional iron injection in the suckling period. Two-iron injection (2): 200 mg iron injections at 2 to 3 d of age and 5 d after the first injection. *n* in parenthesis of the figure represents the number of branches in the tree.

**Figure 4.**
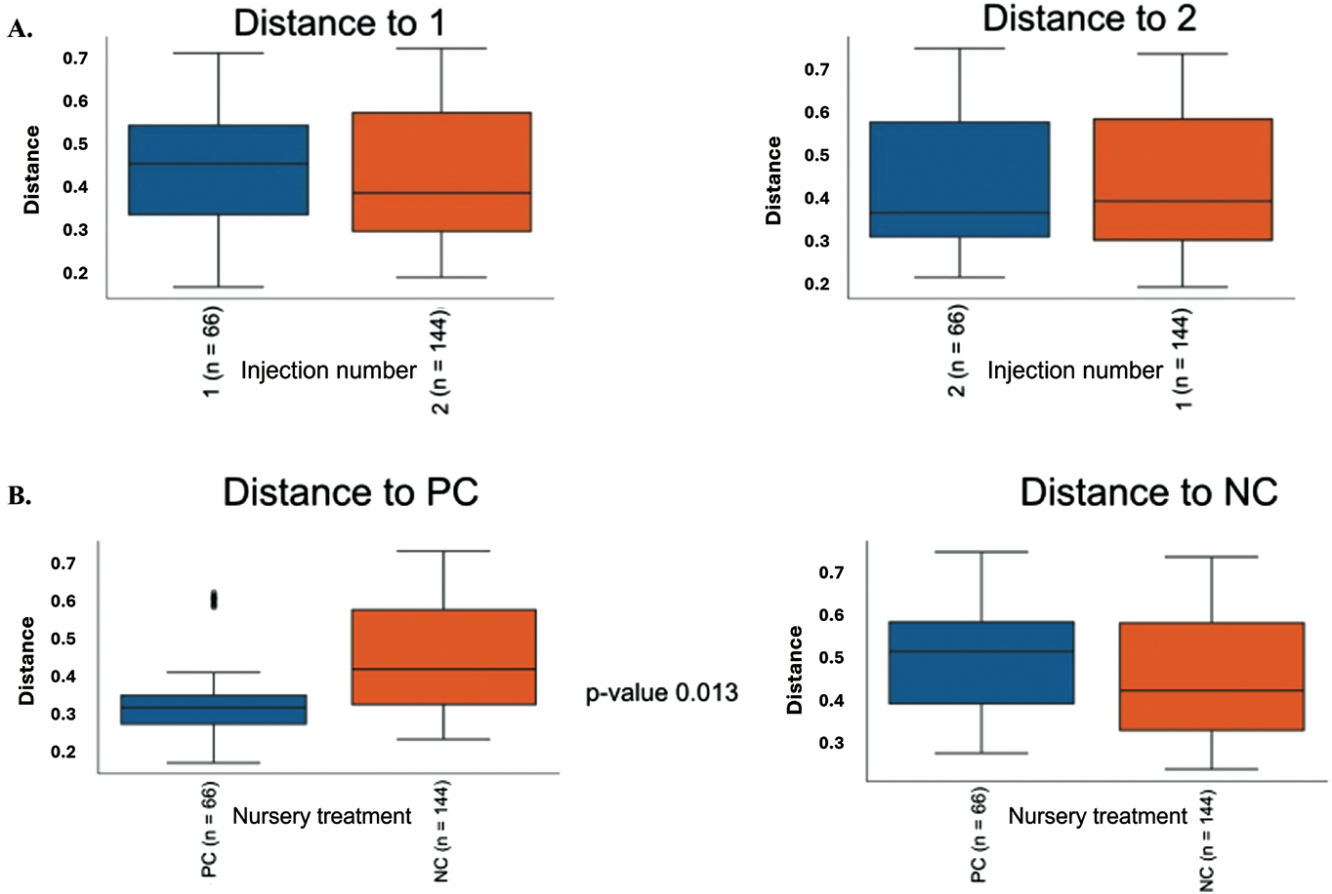
Beta-diversity (Unweighted Unifrac analysis) in feces of pigs at day 27 postweaning. Iron injection effect (A, *P* = 0.59; *n* = 12 per injection treatment): One-iron injection (1): 200 mg iron injection at 2 to 3 d of age and no additional iron injection in the suckling period. Two-iron injection (2): 200 mg iron injections at 2 to 3 d of age and 5 d after the first injection. Dietary iron level effect (B, *P* < 0.05; *n* = 12 per nursery treatment). NC: negative control with 100 mg/kg iron in nursery diets; PC: positive control with 200 mg/kg iron in nursery diets. *n* in parenthesis of the figure represents the number of branches in the tree.

**Figure 5.**
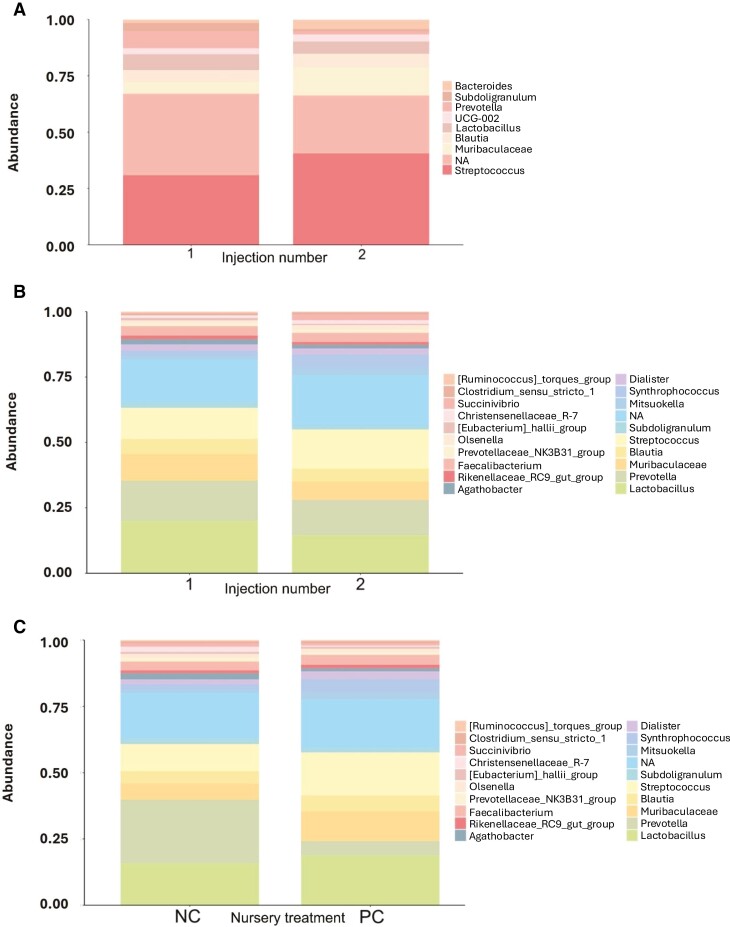
Relative abundance plots in feces of pigs at genus level. Iron injection effect at weaning (A; *n* = 5 per injection treatment) and Iron injection effect at day 27 postweaning (B; *n* = 12 per injection treatment): One-iron injection (1): 200 mg iron injection at 2 to 3 d of age and no additional iron injection in the suckling period. Two-iron injection (2): 200 mg iron injections at 2 to 3 d of age and 5 d after the first injection. Dietary iron level effect at day 27 postweaning (C; *n* = 12 per nursery treatment): NC: negative control with 100 mg/kg iron in nursery diets; PC: positive control with 200 mg/kg iron in nursery diets.

For fecal samples, a total of 612,842 raw reads were obtained from fecal samples from 10 and 24 animals at weaning and day 27 postweaning, respectively. After quality control, combining paired-end reads, and filtering chimeras, on average, 88% of sequences passed the filters. The second iron injection altered the alpha-diversity of the bacterial community in the feces at weaning. Although Evenness was not different between iron injection treatments at weaning (Figure 1), the Faith’s phylogenetic diversity showed that the second iron injection decreased the alpha-diversity in the feces of piglets at weaning (*P* < 0.05; Figure 2). Dissimilarities (*P* < 0.05) in fecal microbial community were observed between 1- and 2-iron injection treatments at weaning (Figure 3) with no difference at day 27 postweaning (Figure 4A) and between dietary iron levels at day 27 postweaning (Figure 4B) based on the beta-diversity with Unweighted Unifrac analysis. Regarding the relative abundance of the fecal microbiome of pigs at the genus level at weaning (Figure 5A), the 2-iron injection treatment showed a higher relative abundance of *Streptococcus*, *Muribaculaceae,* and *Bacteroides* but a lower relative abundance of *Lactobacillus* and *Prevotella* than the 1-iron injection treatment. At day 27 postweaning, the 2-iron injection treatment showed a higher relative abundance of *Streptococcus*, *Mitsuokella*, and *Syntrophococcus* but a lower relative abundance of *Ruminococcus torques* group, *Lactobacillus,* and *Muribaculaceae* than the 1-iron injection treatment (Figure 5B), while the high dietary iron level showed a higher relative abundance of *Lactobacillus*, *Muribaculaceae*, *Streptococcus*, *Mitusuokella*, and *Syntrophococcus* but a lower relative abundance of *Prevotella* (Figure 5C).

## Discussion

The current study evaluated the effect of second iron injection given to suckling pigs during the suckling period on postweaning growth performance, hematological parameters, fecal characteristics, metabolites, and microbiome, along with liver and serum trace mineral content, when pigs were fed nursery diets with or without additional iron supplementation. Due to limited iron supply through the placenta and milk that is associated with a decline in hemoglobin level near weaning, suckling pigs may need an additional iron injection to meet their iron needs in the late suckling and early nursery periods (Chevalier et al., 2021, 2023; Albers et al., 2022). However, dietary iron content from calcium phosphate and limestone and additional iron supplementation using inorganic sources such as ferrous sulfate may exceed the iron requirement estimates for nursery pigs (NRC, 2012). This may result in a high level of unabsorbed iron remaining in the intestine, causing pathogenic bacteria proliferation because *E. coli* and *Salmonella* require iron for their abundance and virulence (Guo et al., 2017; Domínguez-Acuña and García-del Portillo, 2022). Reducing the free iron level in the gut while still maintaining sufficient body iron status with an additional iron injection may be beneficial for pigs to reduce postweaning gut health issues such as diarrhea and improve postweaning growth performance. In the current study, piglets were naturally challenged with rotavirus and *E. coli* within the first week after weaning, as confirmed by the ISU-VDL. These enteric pathogens are common in weaning pigs, resulting in postweaning diarrhea, and therefore, the response of nursery pigs to treatments in the current study was under the condition of enteric disease challenge. In addition, since there were virtually no interactions observed, the discussion focused on the main effects of iron injection and dietary treatments.

In the preweaning period, although the second iron injection increased hemoglobin and hematocrit levels, there was no significant difference in the BW of piglets until weaning. This result agrees with previous studies that showed no difference in preweaning growth performance, although an additional iron injection to suckling pigs increased hemoglobin and hematocrit levels in the suckling period (Albers et al., 2022; Chevalier et al., 2023; Lima et al., 2024). In addition, over 50% and 70% of piglets in the 1-injection treatment had anemic (<9.0 g/dL) and suboptimal hemoglobin levels (<11.0 g/dL) at weaning, respectively, while no piglets had suboptimal hemoglobin levels when receiving the second iron injection. Chevalier et al. (2021) reported that it may take several days for injected iron to be incorporated into hemoglobin compounds. Our previous studies (Albers et al., 2022; Lima et al., 2024) also reported that the response of pigs to the second iron injection for growth performance may not appear until the nursery period. Although the second iron injection in the suckling period did not show any growth performance response in the suckling period, the pigs receiving 2 iron injections during the suckling period tended to have greater BW at day 27 postweaning and growth rate in late and overall nursery periods with 10% difference in BW at day 27 postweaning. This resulted from increased feed intake throughout the nursery period. Lima et al. (2024) investigated various iron injection strategies and reported that the second or split injection to suckling pigs could improve hemoglobin levels and postweaning growth performance. Bhattarai and Nielsen (2015) reported that hemoglobin levels at weaning were positively correlated with postweaning growth rate, which agreed with the current study. As there was no difference in feed efficiency between iron injection treatments, the second iron injection may enhance the robustness of pigs after weaning by improving oxygen transport, immune function, vitality, and intestinal health due to increasing hemoglobin levels, resulting in increased feed consumption and thus the growth rate of pigs (Bhattarai and Nielsen, 2015). Chevalier et al. (2023) reported that the second iron injection to suckling pigs enhanced postweaning growth performance, which was maintained until the pigs reached the market weight. Pierce et al. (2024) reported that a second iron injection upregulated the expression of Claudin 1, and genes associated with epithelial cell proliferation in the duodenum as well as genes involved in vitamin D metabolism in the liver. It also downregulated the expression of genes related to gluconeogenesis and lipid synthesis in the duodenum tissue and urea synthesis in liver, which may lead to substantial energy savings that can contribute to improved growth rates in piglets receiving the second iron injection. However, due to limited number of replication pens used in this study, further study is needed in a larger scale having greater number of pigs and pens and in a commercial setting.

Dietary iron levels did not affect postweaning growth performance during the entire experimental period. Rincker et al. (2004) reported that an additional 100 ppm of iron in the diet could improve the postweaning growth rate of pigs. However, no difference in growth performance in the current study may indicate that an additional 100 ppm of iron supplementation over the requirement might not be high enough to show any difference in growth. This result may be attributed to no difference in hemoglobin levels from weaning to day 13 postweaning between 2 dietary iron levels, as hemoglobin status at weaning is positively correlated with postweaning growth performance (Bhattarai and Nielsen, 2015). Combining with the enhanced growth rate by the second iron injection, it can be noted that increasing the hemoglobin level of pigs before or early after weaning rather than supplementing more iron to the diets may be more effective in enhancing postweaning growth, as the effect of dietary iron on increasing hemoglobin levels may not appear in early nursery period. In addition, depending on body iron status, increasing levels of dietary iron over the iron needs of pigs may cause intestinal oxidative stress and possibly pathogenic bacteria proliferation (Li et al., 2016), as high body iron status could increase hepcidin expression, which in turn reduces iron absorption (Szudzik et al., 2018; Pierce et al., 2024) resulting in high iron content in the gut. Therefore, further study is needed to demonstrate the effect of a second dose of iron with a high dietary iron level for nursery pigs on intestinal oxidative stress and the proliferation of pathogenic bacteria in the early nursery period.

At weaning, although there was no difference in serum trace mineral content, the second iron injection tended to increase liver iron and calcium levels. In addition, the second iron injection increased the serum iron level in the early nursery phase. Chevalier et al. (2021, 2024) also reported similar results in which a high dose of iron administration or second iron injection increased liver deposition of iron in suckling pigs, as there is no such mechanism to remove absorbed iron from the body other than blood loss in mammals (Abbaspour et al., 2014; Wallace, 2016). Therefore, along with hematological parameters, these results indicate that iron injection could increase iron stores in the liver and blood circulation of iron.

Although there were inconsistent responses, the second iron injection and dietary iron levels affected serum levels of a few minerals, mainly divalent cations such as molybdenum, copper, selenium, cobalt, and chromium, as well as phosphorus and potassium, in various postweaning days. When piglets have enough iron in the body, hepcidin expression increases, thus reducing iron absorption, which results in an increased level of unabsorbed iron in the intestine (Lipiński et al., 2010), affecting the absorption of other divalent cations (Malesza et al., 2022). However, further investigation is needed as it is unclear how the second iron injection and dietary iron levels affect blood mineral levels after weaning due to the inconsistent effects of iron injection and dietary iron treatments on serum trace minerals.

In the complete blood count analysis for pigs at weaning, all measurements were within the reference range (Merck, 2016). As expected, the second iron injection increased hemoglobin and hematocrit levels, along with other hematological parameters such as MCV, MCH, and MCHC (except at weaning) until day 13 postweaning. This increase in hematological parameters is expected as piglets did not have an optimal level of hemoglobin (11 g/dL) until weaning with 1 iron injection, and agreed with previous studies (Albers et al., 2022; Chevalier et al., 2023; Lima et al., 2024). Then, the effect of the second iron injection on these parameters was not significantly different from the 1 iron injection at day 27 postweaning, followed by increased red blood cell count, hemoglobin, and hematocrit levels due to the high dietary iron level containing an additional 100 ppm of iron in nursery diets. This change may be attributed to increased BW during 27 d of nursery period that increases blood mass of the animals (Pettey et al., 2015). Similarly, Chevalier et al. (2023) reported that a second iron injection maintained significantly higher hemoglobin levels in pigs than 1 iron injection until the end of the nursery period (5 wk after weaning), but the magnitude of the difference declined from 2.4 g/dL at weaning to only 0.4 g/dL at 5 wk postweaning. This result indicates that when the second iron injection was performed in early suckling period, the effect on increasing hematological parameters can be maintained until a few weeks after weaning, at least in the early nursery period. Thus, the high level of iron in the diets for early nursery phase may be redundant if the second iron injection was performed in the suckling period in terms of iron needs for the body.

Interestingly, piglets receiving 1 iron injection had greater platelet count and plasma protein levels until day 13 postweaning compared with those with 1 iron injection. It has been previously reported that iron deficiency anemia may cause an increase in platelet count in the body as an adaptive biological response to counter potential blood loss for survival instinct, and iron treatment in human patients with iron deficiency anemia may decrease platelet count (Li et al., 2022). 50% of piglets in the 1-injection treatment became anemic at weaning in the current study and this may result in an increased platelet count. Further study is needed to demonstrate how iron status affects platelet count and plasma protein levels in pigs.

Regarding the fecal microbiome, 2 iron injections lowered the alpha-diversity based on Faith’s phylogenetic diversity at weaning compared to 1 iron injection, while there was dissimilarity in fecal bacterial communities at weaning between 1- and 2- iron injection treatments based on the Unweighted Unifrac analysis. There is limited information regarding gut microbiome change by different strategies of iron injection to suckling pigs. La Carpia et al. (2019) reported that intravenous iron infusion and iron status could modify gut microbiota in mice. Therefore, the change in body iron status by the second iron injection may influence gut microbiome. This has also been confirmed by the result of the fecal score, in which the second iron injection reduced the fecal score in the nursery period, which indicates firmer feces and improvement of the gut environment. However, as there is limited information regarding how iron status or iron injection strategies alter gut microbiome, further study is needed to demonstrate the effect of iron administration routes on gut microbiome in pigs.

Dietary iron level altered the fecal microbial community, where there was dissimilarity in the fecal bacterial community at day 27 postweaning based on Unweighted Unifrac analysis. Dietary iron has a significant effect on gut microbiota as iron is required for pathogenic bacteria proliferation, and free iron may increase oxidative stress in the gut (Li et al., 2016; Domínguez-Acuña and García-del Portillo, 2022). When inorganic iron is orally supplemented, it could increase the abundance of *E. coli*, and Proteobacteria but decrease *Prevotella*, *Lactobacillus*, and *Bifidobacterium* and possibly increase the risk of diarrhea and enteric infection (Abbas et al., 2022). Although there was a slight reduction in fecal score indicating slightly firmer feces in the late nursery period when the high level of dietary iron was fed to pigs, the fecal score was not significantly different in overall period. Therefore, these results indicate that the changes in gut microbiome and environment caused by an additional 100 ppm of iron supplementation to nursery diets over the iron requirement (100 ppm) may not be sufficient to impact postweaning growth performance, although it should be noted that an increased dietary iron level could increase hemoglobin and hematocrit levels in the late nursery period.

In the result of the abundance of fecal microbiome at genus level, the second iron injection decreased the relative abundance of *Prevotella* and *Lactobacillus* at weaning, *Lactobacillus*, and *Ruminococcu torques* group at day 27 postweaning, while increasing the relative abundance of *Bacteriodes* and *Streptococcus* at weaning and *Streptococcus* at day 27 postweaning. *Prevotella* and *Lactobacillus* are found in the healthy preweaning microbiota of pigs (Jenkins et al., 2024). The relative abundance of *Ruminococcus torques* group and *Lactobacillus* increased by *Lactobacillus rhamnosus* GG probiotic supplementation to weaning pigs (Bai et al., 2022). However, although the second iron injection increased the relative abundance of *Streptococcus*, which was found to be higher in pigs growing faster as a potential probiotic candidate (Wang et al., 2019; He et al., 2023), the relative abundance of beneficial bacteria genera was reduced at weaning and in the nursery period by the second iron injection. Knight et al. (1983) reported that a higher level of iron injection to suckling pigs might increase their susceptibility to bacterial infection due to their high serum iron content and the second iron injection reduced microbial richness in the current study. Although the second iron injection increased postweaning growth performance, it is still unclear how the additional iron injection in the suckling period alters the fecal microbiome and how this change in the fecal microbiome affects postweaning growth. The high dietary iron level treatment showed a lower relative abundance of *Prevotella* but a higher relative abundance of *Lactobacillus* and *Streptococcus* in the late nursery period than the low dietary iron level treatment. Chen et al. (2020) reported that increasing dietary iron levels up to 800 ppm for weaning pigs increased diarrhea incidence while also increasing the relative abundance of *Lactobacillus* in colonic digesta. Due to inconsistent results in the relative abundance of bacterial genera in the current study, further study is warranted to investigate how body iron status and dietary iron level affect gut microbiota and the environment, thus affecting postweaning growth.

As dietary iron levels and body iron status that affect iron absorption from the gut altered the fecal microbial community in the current study, we hypothesized that those factors may have the potential to influence short-chain fatty acid production in the gut. However, iron injection and dietary iron level did not change fecal short-chain fatty acid concentrations at day 27 postweaning in the current study. An iron-deficient diet fed to rats resulted in significantly lower butyrate and propionate concentrations in the cecal digesta compared with an iron-sufficient diet, but excess iron may not have a notable impact on the gut microbiota compared with iron deficiencies (Dostal, et al., 2012; Rusu et al., 2020). Therefore, the levels used for additional iron injection and supplementation in the current study may not lead to an alteration of fecal metabolites, although those influenced the fecal microbiome.

In conclusion, the second iron injection given to suckling pigs improved postweaning growth performance and the levels of hemoglobin and other hematological parameters, reduced fecal score indicating firmer feces and platelet count, and affected serum mineral levels, white blood cell counts, and the fecal microbiome community. An additional 100 ppm of dietary iron over the requirement estimate increased hemoglobin levels, reduced fecal score, and altered white blood cell counts and the fecal microbiome community in the late nursery period but did not affect postweaning growth. The current study also demonstrated that the effect of the second iron injection on hematological parameters could be maintained high until the first few weeks postweaning, while its effect on growth performance could be observed in the late nursery period, although the second iron injection is performed in the early suckling period.

## Abbreviations

ADFI: average daily feed intake
ADG: average daily gain
BW: body weight
EDTA: ethylenediaminetetraacetic acid
G:F: gain-to-feed
i.m.: intramuscular
MCH: mean corpuscular hemoglobin
MCHC: mean corpuscular hemoglobin concentration
MCV: mean corpuscular volume
NRC: National Research Council
SCFA: short-chain fatty acid
SID: standardized ileal digestible
STTD: standardized total tract digestible
